# Dermatology

**Published:** 1892-12

**Authors:** 

**Affiliations:** Dermatologist to Charity Hospital and Lecturer in Tulane Medical College, New Orleans, La.


### Dermatology.

[Edited by ISADORE DYER, M. D., Dermatologist to Charity Hospital and Lecturer in Tulane Medical College, New Orleans, La.]

---

### NOTES ON DERMATOLOGY.

The foreign journals of dermatology for September and October are filled with the recent convention held in Vienna. The better papers are reported in full with their discussion. The French school seem to have provided the most of the original work, and Drs. Besmer and Brocq, of Paris, and Dr. Leloir, of Lisle, carried the honors. Following the exhaustive discussion of erythematous lupus at the convention comes Dr. Henri Leloir's work on the "Scrofulo-Tuberculosis" of the skin. Of this work Dr. Fournier has given an excellent review in his *Journal des Maladies Cutaneés et Syphilitique.* Dr. Leloir holds erythematous lupus non tubercular. He moreover suggests that true lupus vulgaris is present early or late in such cases of erythematous lupus where the tubercle bacillus is found. He confesses to having made this error in diagnosis himself on account of the intimate resemblance in the objective symptoms. Dr. Leloir divides skin tuberculoses into primary and secondary groups for the more definite prognosis and the better pathological investigation. Lupus of the colloid and myxomatous types, with the vascular types, belong to the primary class. Cicatrizing lupus, with erythematoid lupus (resembling lupus erythematosus) are embraced in the secondary group. To these add the *gommes tuberculeuses*, the ulcerating tuberculosis cutis, tubercular glands, and the divisions are complete. The author accepts the conclusion that in lupus constitutional symptoms may be present, and that lupus can be found in combination with other forms of tuberculosis of the skin. The work in the main is characterized by the usual careful study and pathological work of the author.

---

Dr. Buret, contributing to the same journal, presents an interesting argument for the existence of syphilis or some venereal disease in the ruined cities of Herculaneum and Pompeii. His belief and his article are based on the study of certain frescoes and legends on stone, preserved by the Italian government in the museum at Naples. The effort should be appreciated by us as one tending to lift still more conclusively from America the onus of the origin of syphilis.

---

The strepto-bacillus of the soft chancre, first discovered and reported by Dr. P. G. Unna, of Hamburg, is creating quite a commotion in dermatological circles. If accepted, it means an absolute differentiation between chancres and chancroids, and, perhaps, will lead to the remedy, which, acting specifically, will mean the extermination of the chancroid. At first dubiously viewed in the French Dermatological Society, Quinquan alone accepting Unna's work, it has become a recognized member of the microbe group, and is now bringing honor to the discoverer. In a fresh chancroid the streptococcus is readily found, but only in the lymph spaces between the cellules of the tissue diseased. Cultures are best made from the tissues themselves, rather than from the discharges. This streptococcus has not been found in other ulcerations. It is distinguished from other micro-organisms by qualities peculiarly its own and which have been found capable of reproducing the conditions which characterize the soft chancre. The organism is a true streptococcus, of short rod development, from ¼ m. to 3 m. in in length.

---

Dr. Boeck, of Christiana, suggests, in treating lupus vulgaris, the simultaneous use of nitrate of silver and iodoform. In this way, he claims, some chemical action occurs, evidenced by a bubbling or frothing at the spot treated. A happy therapeutic result is said to be obtained. The proceeding consists in first scraping the lesions; after three days the ulcerating points are cauterized with the silver stick, the caustic cutting well into the lesions. At once a ten per cent. iodoform in collodion pigment is painted on. This is repeated daily, and success is reported.

---

Saalfield reports successful removal of freckles, chloasma and vitiligo lesions with one per cent. of bichloride of mercury solution applied on compresses. The treatment is excellent, but the patient must be warned of the inflammation and vesication to follow. A ten per cent. ichthyol ointment with one drachm of carbonate of magnesia added will relieve this condition. One-half of one per cent. solution of bichloride in collodion, painted on the lesions, will accomplish the same purpose with less discomfort to the patient.

---

The seventh part of the "International Atlas of Rare Skin Diseases" has just appeared with three excellent plates and corresponding articles from the pens of Dr. Ernest Besnier, of Paris, Chronic Perforating Farcy; Drs. G. Lewin and J. Heller, of Berlin, on Cutaneous Syphilitic Pains, and Dr. H. G. Brooke, of Manchester, England, on Contagious Follicular Keratosis.

The Atlas possesses the advantage of being published in English, German and French, and the press of Leopold Vass, in Hamburg, guarantees the accuracy of the plates.

Dr. Brooke's article is especially interesting in that the disease is shown to be a contagious one, a fact never before reported, though the disease has been observed ever since Casenave reported it, in 1824, under the name of *acne sebaceè corneé.* Dr. Brooke, with Dr. P. A. Morrow, of New York, accepts the disease as a true keratosis and as originating in the fat gland or hair follicle. The contagious nature is evidenced by the spread of the disease to contiguous parts and from one member to others of the same family. The "psorosperm" is probably the source of contagion but Dr. Brooke is not prepared to assert himself on this point. The disease consists of a primary papular eruption, brownish in color, undergoing gradual changes until the papules become regular spines on the skin. The spines, some quite long, are exceedingly elastic, strong, and horny in structure. They develop directly from a hair follicle. The distribution is general and systematical though worse on the extremities. The disease begins usually on the neck and spreads up and down, until the forehead, face, arms, chest and legs are affected. The treatment of the eight cases reported consisted in the free application of emolients, which readily softened the processes and relieved the conditions.

